# Lady Doctors in Germany

**Published:** 1898-02-05

**Authors:** 


### LADY DOCTORS IN GERMANY.

Although the German Universities do not as yet grant medical degrees to women, it is announced that the Chancellor is about to open the State examination to them, and as this examination is the one portal to the legal practice of medicine in Germany, and has to be undergone even by members of the Universities, the women's right to practise in that country will be fully secured.

